# Predicting the brain age of children with cerebral palsy using a two-dimensional convolutional neural networks prediction model without gray and white matter segmentation

**DOI:** 10.3389/fneur.2022.1040087

**Published:** 2022-11-24

**Authors:** Chun-yu Zhang, Bao-feng Yan, Nurehemaiti Mutalifu, Ya-wei Fu, Jiang Shao, Jun-jie Wu, Qi Guan, Song-hai Biedelehan, Ling-xiao Tong, Xin-ping Luan

**Affiliations:** Cerebral Palsy Center in Neurosurgery, Second Affiliated Hospital of Xinjiang Medical University, Urumqi, China

**Keywords:** cerebral palsy, brain age, deep learning, convolutional neural networks, brain age gap estimation

## Abstract

**Background:**

Abnormal brain development is common in children with cerebral palsy (CP), but there are no recent reports on the actual brain age of children with CP.

**Objective:**

Our objective is to use the brain age prediction model to explore the law of brain development in children with CP.

**Methods:**

A two-dimensional convolutional neural networks brain age prediction model was designed without segmenting the white and gray matter. Training and testing brain age prediction model using magnetic resonance images of healthy people in a public database. The brain age of children with CP aged 5–27 years old was predicted.

**Results:**

The training dataset mean absolute error (MAE) = 1.85, *r* = 0.99; test dataset MAE = 3.98, *r* = 0.95. The brain age gap estimation (BrainAGE) of the 5- to 27-year-old patients with CP was generally higher than that of healthy peers (*p* < 0.0001). The BrainAGE of male patients with CP was higher than that of female patients (*p* < 0.05). The BrainAGE of patients with bilateral spastic CP was higher than those with unilateral spastic CP (*p* < 0.05).

**Conclusion:**

A two-dimensional convolutional neural networks brain age prediction model allows for brain age prediction using routine hospital T1-weighted head MRI without segmenting the white and gray matter of the brain. At the same time, these findings suggest that brain aging occurs in patients with CP after brain damage. Female patients with CP are more likely to return to their original brain development trajectory than male patients after brain injury. In patients with spastic CP, brain aging is more serious in those with bilateral cerebral hemisphere injury than in those with unilateral cerebral hemisphere injury.

## Introduction

Cerebral palsy (CP^1^) is a clinical syndrome characterized by dyskinesia and abnormal posture resulting from non-progressive but irreversible damage caused by cerebral ischemia and hypoxia during fetal brain development (1). The incidence of CP in children has gradually exceeded 3 per 1,000 (1, 2). Globally, there is a relatively large number of patients with CP. Currently, rehabilitation is the primary treatment for CP (3), and depending on surgical indications, some children may undergo selective posterior rhizotomy (4) (SPR^2^) or cervical perivascular sympathectomy (5) (CPVS^3^). Previous studies on CP have mainly focused on early diagnosis, brain injury characterization, and neurodevelopmental outcomes in newborns with CP (6–8). There is currently no report on the pattern of brain development with disease progression in children with CP.

With regard to the description of brain development in patients with CP, it is more common for researchers to find white matter damage and gray matter atrophy in patients with CP through magnetic resonance image (9) (MRI^4^), but it does not specify the exact brain age of patients with CP after brain atrophy. It is hypothesized that the trajectory of brain development of patients with CP changes following this kind of brain damage.

The three-dimensional convolution neural network (3D-CNN^5^) brain age prediction model is a good predictor of brain age (10, 11). As a physiological index, the brain age gap estimation (BrainAGE^6^) (12, 13) method can effectively predict the degree of brain development in patients with neuropsychiatric disorders (12, 14, 15). However, the theoretical basis of these models is contingent on the volume and proportion of white and gray matter during the development of the human brain. It is necessary to segment the white and gray matter in the process of magnetic resonance preprocessing. Our hospital has accumulated a large number of magnetic resonance data for the diagnosis and treatment of CP. Because the voxels of children with CP in our hospital do not meet the basic requirements of the Montreal Neurological Institute (MNI^7^) template, we cannot clearly segment the white and gray matter, and thus, we cannot obtain the white matter map and gray matter map. Additionally, we cannot use the 3D-CNN brain age prediction model to predict the brain age of children with CP. The establishment of the two-dimensional convolution neural network (2D-CNN^8^) brain age prediction model provides the possibility to solve this problem (16, 17). However, because the training of the model is still based on the input of white and gray matter images, it cannot meet the demand.

To effectively use these MRIs^9^ to study brain development in children with CP, a 2D-CNN brain age prediction model that can avoid segmenting white and gray matter was designed, and a retrospective cohort study was conducted using existing MRIs to explore the pattern of brain development in children with CP aged 5–27 years old.

## Materials and methods

### Participants

We downloaded the head MRI of 3,735 healthy humans from 14 public datasets (male/female = 2,128/1,607, mean age = 26.93 ± 19.03, age range = 5–86 years) (10, 18–20). The images in the dataset are from various locations, and the instruments and parameters employed vary, ensuring the universality of the model (SupplementaryTable S1). Permission to download and use all datasets was obtained before conducting data analysis. The local ethics committee ethically approved all projects in the datasets, and all healthy participants signed informed consent forms at the scanned sites. According to the local research plan, all healthy controls in the dataset had no diagnosis of neurological or mental illnesses when collecting the MRI scans.

The head MRI of 667 patients with CP (male/female = 387/280, mean age = 7 ± 3.97, age range = 1–27 years) came from the CP center of the second affiliated hospital of Xinjiang Medical University, which is the only hospital designated by the Ministry of Civil Affairs in Northwest China for CP surgery.

The costs of examination and treatment of children with CP were borne by the Ministry of Civil Affairs. This study was approved by the ethics committee of the second affiliated hospital of Xinjiang Medical University. Written informed consent was abandoned because this study was a retrospective study.

### MRI screening

The MRI of healthy people left 3,474 cases (male/female = 2,035/1,439, mean age = 25.39 ± 18.20, age range = 5–86 years) after excluding artifacts and images with abnormal brain MRI findings. After excluding patients under the age of 5 and all patients with MRI artifacts, there were 455 patients left (male/female = 272/183, mean age = 8.71 ± 3.70, age range = 5–27 years). Patients with CP were examined using a Philips 1.5T scanner. The T1-weighted sequences scanning parameters were as follows: TE = 15 ms, TR= 487 ms, flip angle = 90°, matrix = 256 × 163 mm, slice thickness = 6 mm, slices = 18, FOV = 230 × 183 × 118 mm, voxel = 0.9 × 1.12 × 6 mm.

### Data preprocessing

(1) We used dcm2niix (https://github.com/rordenlab/dcm2niix) to convert the MRI data of cerebral palsy patients (DICOM) to NIfTI format. Since the MRI data in the public datasets are already in NIfTI format, no further format conversion is required; (2) based on the clinical data acquisition parameters, we used the linear registration flirt of the FMRIB Software Library (FSL) (https://fsl.fmrib.ox.ac.uk/fsl/fslwiki) to align all data to the MNI152 1-mm standard T1 structure and resampled to 128 × 128 × 18, voxel size = 1.42 × 1.70 × 10.11 mm. (3) Since the rigid transform only translates and rotates the image without changing the structure of the brain itself, preserving enough individual differences for feature extraction by the convolution kernel, we use the rigid transform to align all data to the standard template and resample them as above; (4) Due to differences in acquisition parameters and equipment, to ensure uniform measurements, all data are standardized by *z*-score.

### Construction of the brain age prediction model

#### The framework of the brain age prediction model

**Figure 1:**
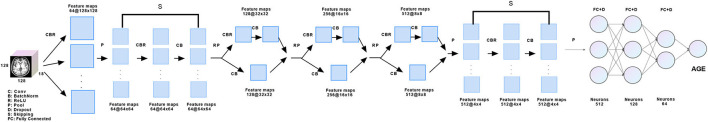
Model schematic. At the beginning of the model, a 2D convolution layer composed of three convolution kernels was used for feature extraction, followed by a skipping layer composed of nine skipping blocks to capture deeper features. Features were learned again through three groups of convolution layers designed by reference GoogLeNET, and the same skipping layer was used to capture deeper features. Finally, the brain age was regressed through the full connection layer.

A small number of skipping blocks was used to extract image features better, while the middle part referred to GoogLeNet to make the network wider, integrate the features of different convolution kernels, and achieve a multi-model fusion effect. Finally, we used three layers of the full connection layer to return to the brain age, and each layer was followed by the Dropout layer to avoid over-fitting. Training strategy: the learning rate is 0.00008, and the optimization strategy is Adam, loss: mean squared error (MSE^10^) (Figure 1).

#### Training and testing of models

**Figure 2:**
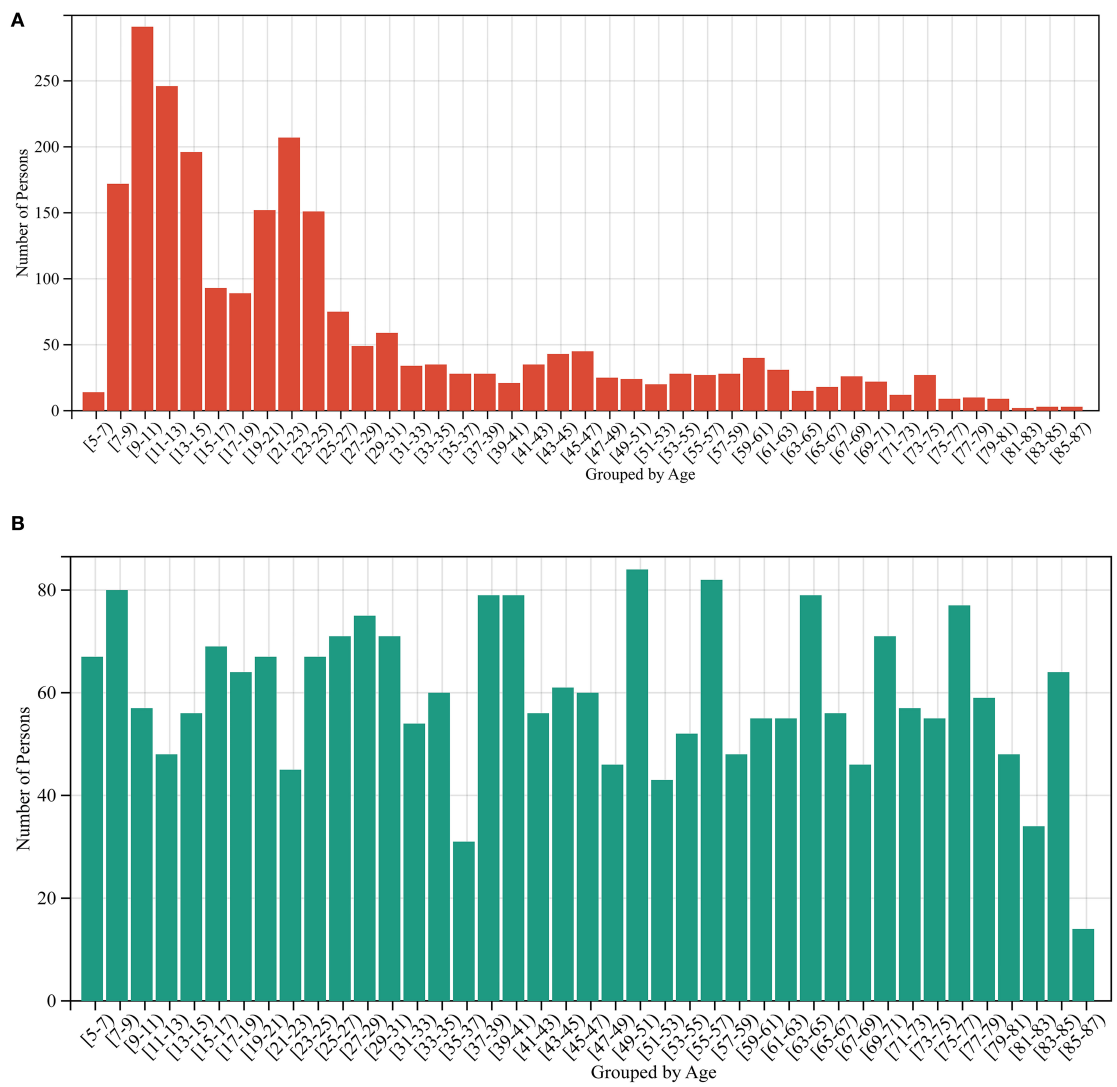
Sample size distribution before and after stratified sampling. **(A)** The sample distribution before stratified sampling, and **(B)** the sample distribution after stratified sampling.

A total of 2,442 out of 3,474 MRI data of healthy people were randomly selected for the training model, and the remaining 1,032 healthy people were used as the test set. The data in the training set are independent of the data in the test set. Due to the uneven age distribution of the healthy people in the training set (Figure 2A), a hierarchical sampling strategy was used for the training set data (Figure 2B). The performance of the predictive model was evaluated using mean absolute error (MAE^11^), correlation coefficient *r*, determination coefficient (*R*^2^^12^), root-mean-squared error (RMSE^13^), and BrainAGE.

### Statistical methods

Kolmogorov–Smirnov test was used to determine whether the data were normally distributed. Normally, distributed data were described using mean ± standard deviation (mean ± SD). The brain age difference between the two sets was compared using an independent samples *t*-test. The brain age and physiological age of patients with CP were compared by paired *t*-test. The chi-square test was used to compare sex and other classified variables. Pearson correlation analysis was used to measure the association between continuous variables. Statistical analyses were performed using SPSS22.0.

## Results

### Effect of the brain age prediction model based on 2D-CNN

**Figure 3:**
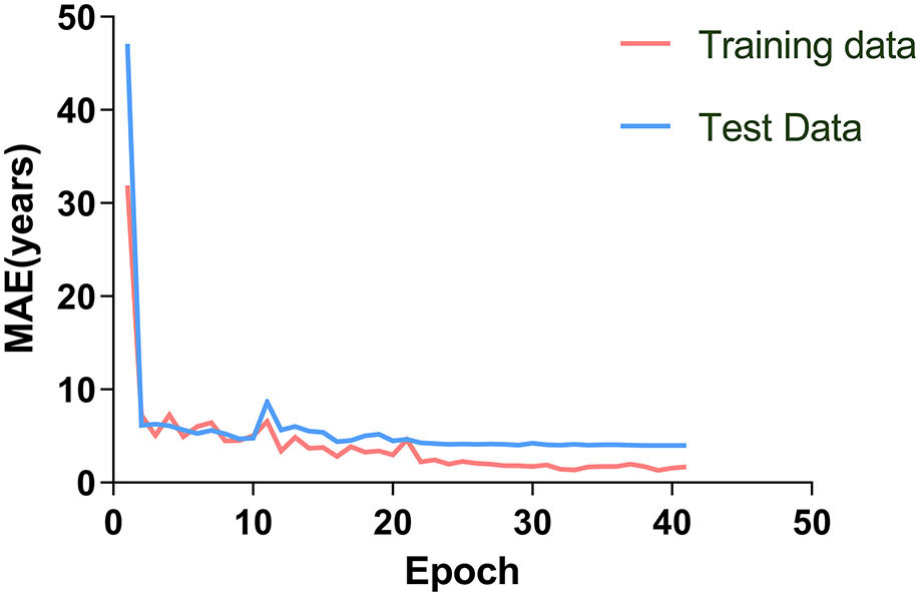
The brain age prediction model was trained 40 times. The MAE of the training and test sets decreased gradually and tended to be stable with increased training times.

**Figure 4:**
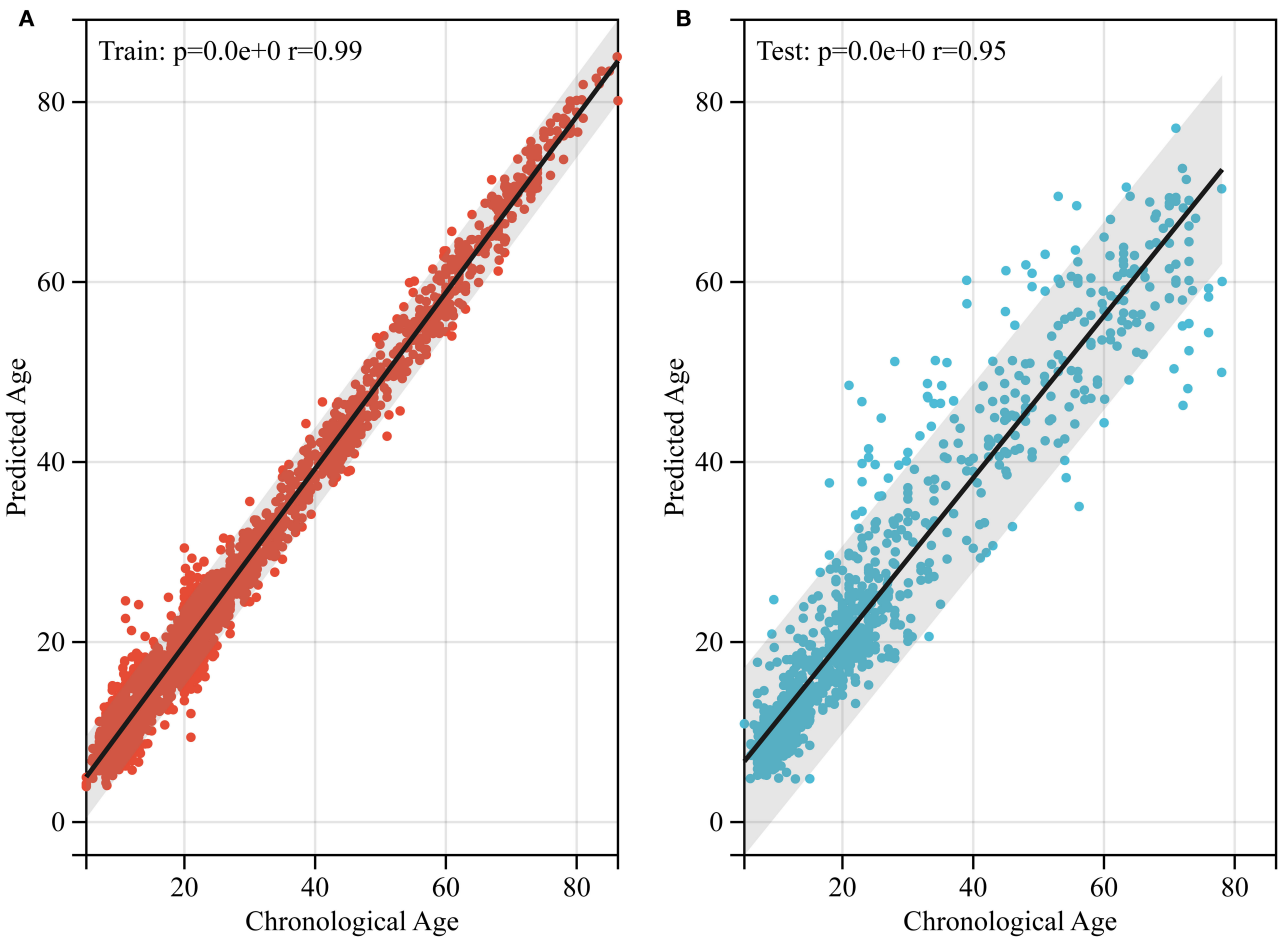
Performance of brain age prediction model. **(A)** The physiological age of the training set and its predicted brain age (*r* = 0.99, *p* < 0.0001). **(B)** The physiological age of the test set and its predicted brain age (*r* = 0.95, *p* < 0.05).

Without segmenting white and gray matter, our 2D-CNN model accurately predicted the brain age of healthy people using MRI after simple preprocessing (Table 1, Figures 3, 4).

**Table 1:** Performance of the brain age prediction model.

	**MAE**	**RMSE**	***R***	***R^2^***
Train	1.85	2.39	0.99	0.98
Test	3.98	5.6	0.95	0.9

### Data analysis of brain age in patients with cerebral palsy

The BrainAGE data in the CP group were normally distributed (*p* = 0.076). The BrainAGE in the CP group (*n* = 455, 20.28 ± 2.9) was higher than that in the healthy control group (*n* = 442, 0.25 ± 2.90). This difference was found to be statistically significant (*p* < 0.0001). There is a negative correlation between BrainAGE and physiological age in patients with CP aged 5–27 years old (*r* = −0.14, *p* < 0.01).

### Sex comparison

In children with mixed CP, there was no significant difference in the number of asphyxia and non-asphyxia-related cases between male and female patients (χ^2^ = 2.467, *p* = 0.116). In patients with mixed CP, the BrainAGE was higher in male patients (*n* = 96, 20.64 ± 10.20) than in female patients (*n* = 88, 17.17 ± 9.28), and the difference was statistically significant (*p* < 0.05). In summary, sex has an impact on brain age differences in patients with CP.

### Comparison in terms of disease classification

As shown in Table 2, there was a large difference in sample size between the spastic and mixed CP groups, which was not comparable. After excluding the cases where the cause was not determined, the difference in sample size between the two groups was very small. The male to female ratios of the spastic and mixed CP groups did not match upon analysis (χ^2^ = 6.509, *p* < 0.05). The male proportion of patients with spastic CP was higher than those with mixed CP. There was no significant difference in the proportion of asphyxiated and non-asphyxiated patients between the two types of CP (χ^2^ = 3.652, *p* = 0.056). The BrainAGE of female patients with spastic CP (*n* = 85, 21.22 ± 10.57) was higher than that of female patients with mixed CP (*n* = 88, 17.17 ± 9.28), and the difference was statistically significant (*p* < 0.01).

**Table 2:** Clinical data and statistics for cerebral palsy.

**Types of cerebral palsy**	**N**	**Male/female**	**Asphyxia**	**Non-asphyxia**	**Unclear etiology**
Spastic cerebral palsy	240	155/85	108	71	61
Mixed cerebral palsy	184	96/88	65	54	65
Athetoid cerebral palsy	16	12/4	6	5	5
Other types of cerebral palsy	15	9/6	4	4	7

### Analysis from the perspective of etiology

Excluding patients with unknown etiology, there was no significant difference in BrainAGE between asphyxiated (*n* = 26, 15.87 ± 10.73) and non-asphyxiated (*n* = 28, 19.74 ± 6.91) female patients with mixed CP (*p* = 0.12).

### Spastic cerebral palsy

In patients with spastic CP, there was no significant difference in the male-to-female ratios between spastic bilateral and spastic unilateral paralysis (χ^2^ = 0.392, *p* = 0.531). The BrainAGE of patients with bilateral spastic CP (*n* = 111, 23.47 ± 10.81) was higher than that of those with unilateral spastic CP (*n* = 129, 20.62 ± 9.79), and the difference was statistically significant (*p* < 0.05).

## Discussion

The brain age prediction model based on MRI shows considerable performance in predicting the degree of brain development (21, 22). However, not all MRI data can distinguish between white and gray matter. Since the application of MRI in clinical care, it is believed that many hospitals have accumulated a large number of MRI data. However, because the data are not high-resolution imaging, they cannot be applied to scientific research. Scientific research involving a large sample size is often objective and representative of the general population. The brain age prediction model based on 2D-CNN resolves the issues of time cost and small sample size, assists researchers in conducting retrospective studies using existing MRIs, and avoids the challenge of segmenting white and gray matter. Thus, the auxiliary effect of BrainAGE on clinical decision-making of neuropsychiatric diseases can be quickly realized.

Recently, a brain age prediction model based on 2D-CNN was designed. However, the selected age ranges of the model were mostly adults or children aged 0–5 years. The brain age prediction model of 2D-CNN covering children to elderly patients has not been reported. It is known that age width, magnetic resonance sequence selected for modeling, and modeling methods can affect the accuracy of brain age prediction (12, 13, 17, 23, 24). The brain age prediction model is better than the adult prediction model in the prediction performance of healthy children aged 5–18 years. The accuracy of the brain age prediction model was better in patients aged 5–18 years than in patients above 18 years of age. We were unable to obtain the MRI data of healthy children aged 1–5 years due to an issue with public dataset access, thus, we chose children with CP aged 5 and above.

**Figure 5:**
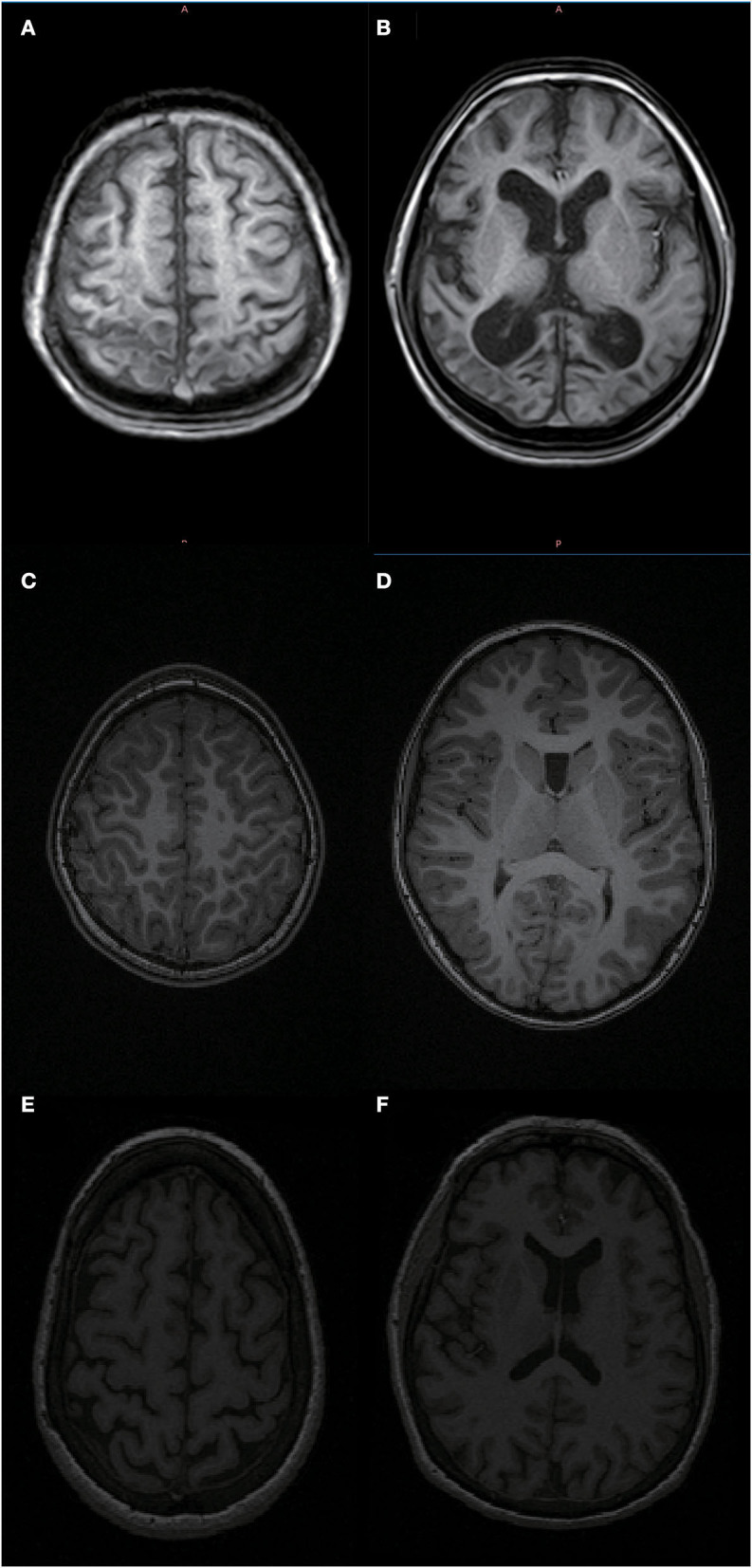
MRI of an 11-year-old patient with bilateral spastic cerebral palsy vs. an 11- and 62-year-old healthy person. As shown in **(A,B)** the head MRI of an 11-year-old patient with bilateral spastic cerebral palsy, who has significant brain atrophy and the patient's brain age is 61.74 years; **(C,D)** the head MRI of an 11-year-old healthy person; **(E,F)** the head MRI of a 62-year-old healthy elderly person. Based on the pictures, we can see that the 11-year-old bilateral spastic cerebral palsy patient's degree of brain atrophy is relatively close to that of a 62-year-old healthy elderly person.

**Figure 6:**
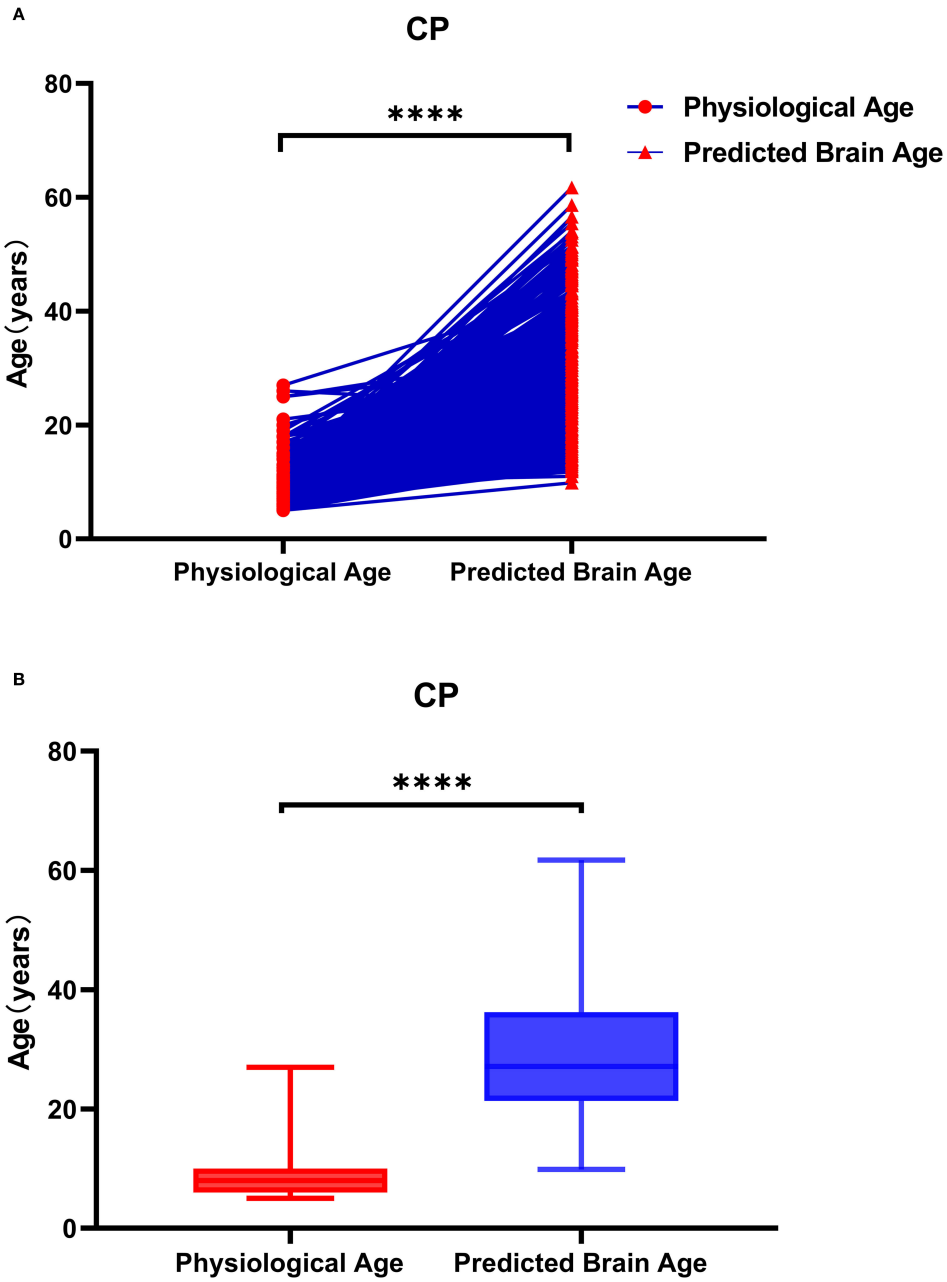
Physiological age and brain age of patients with cerebral palsy. As shown in **(A)**, the data represented by the circle icon are the physiological age of the CP patient, and the triangle icon represents the corresponding actual brain age. As shown in **(B)**, the brain age of patients with CP is older than the physiological age. As shown in **(A)**, the data on the left are the physiological age of patients with CP, and the data on the right are the corresponding actual brain age. The brain age of patients with CP was greater than their physiological age.

After a preliminary observation of the MRI of children with CP, we found that the brain age of children with CP was much older than their physiological age (Figure 5). To fully cover these extreme cases, we chose the age range of the prediction model to be 5–80 years. The results (Figure 6) confirmed our speculation that the brain age of children with CP was much older than their physiological age, and the small-scale brain age prediction model could not fully cover the brain age range of patients with CP. Although the wide age range and limited sample size affected the accuracy of the model, it also successfully revealed the trend of brain development in children with CP. In addition, based on the MAE and *r* values of the model, the performance of the brain age prediction model of 2D-CNN without sectioning the white and gray matter was not significantly different from that of some 3D or even 4D-CNN (10, 24). However, compared to 3D and 4D models, the 2D-CNN models are more difficult to comprehend. Our brain age prediction model may be more suitable for hospitals to conduct a retrospective study of the existing large sample data when the quality of MRI is poor so as to clarify the impact of some neuropsychiatric diseases on the trajectory of brain development.

**Figure 7:**
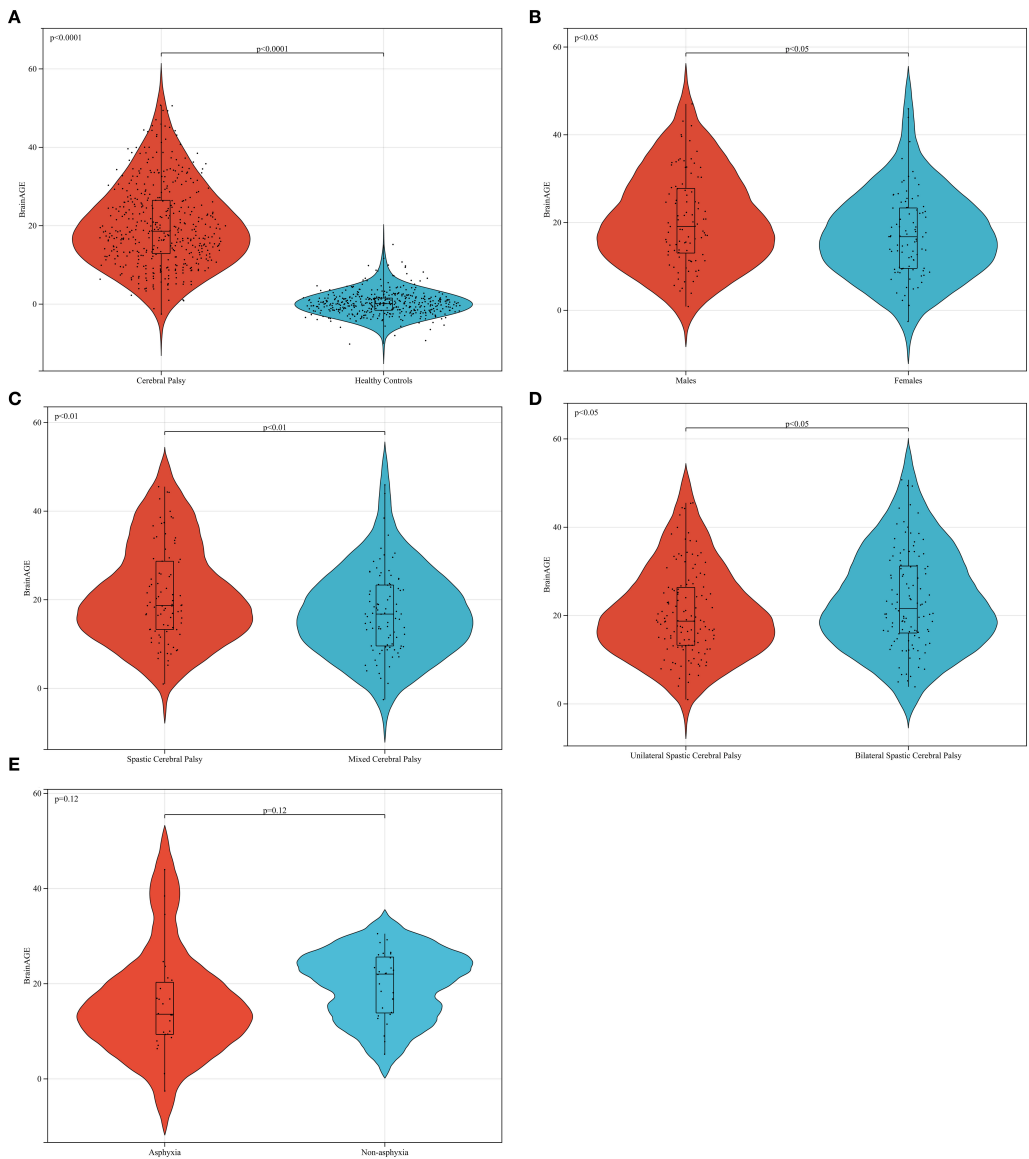
Data analysis of brain age difference in patients with cerebral palsy. In **(A)** the BrainAGE of patients with CP is higher than that of healthy peers (*p* < 0.0001). In **(B)** the brain age difference of men is higher than that of women in patients with mixed cerebral palsy (*p* < 0.05). In **(C)** the brain age difference of female patients with spastic CP was higher than that of patients with mixed CP (*p* < 0.01). Furthermore, the BrainAGE of patients with bilateral spastic CP was higher than that of patients with unilateral spastic cerebral palsy (*p* < 0.05), as shown in **(D)**. In **(E)** the BrainAGE of patients with non-asphyxia was higher than that of patients with asphyxia in female mixed CP (*p* = 0.12).

Figure 7A shows that the brain of the children with CP was aging, consistent with the description of brain atrophy in ~80% of patients with CP on abnormal MRI (7). This is because children with CP suffer from white matter injury, ischemia, hypoxia, intracerebral hemorrhage in fetuses, and a decrease in the gray matter volume after birth (9, 25). Bethlehem et al. found that gray matter reaches its peak during brain development in healthy children at age 6 and then steadily declines; white matter reaches its peak at age 27 and then gradually declines; subcortical matter reaches its peak at age 14 and then gradually declines; the ventricle reaches a small peak at age 5 declines slightly, and then tends to level off, and rises rapidly after age 45 (18). The gray matter of CP children decreases rapidly after birth, and the degree of gray matter decrease is higher than that of white matter reduction, so the brain age of CP children is higher than that of their peers in the human brain map, reaching the level of the middle aged and elderly.

**Figure 8:**
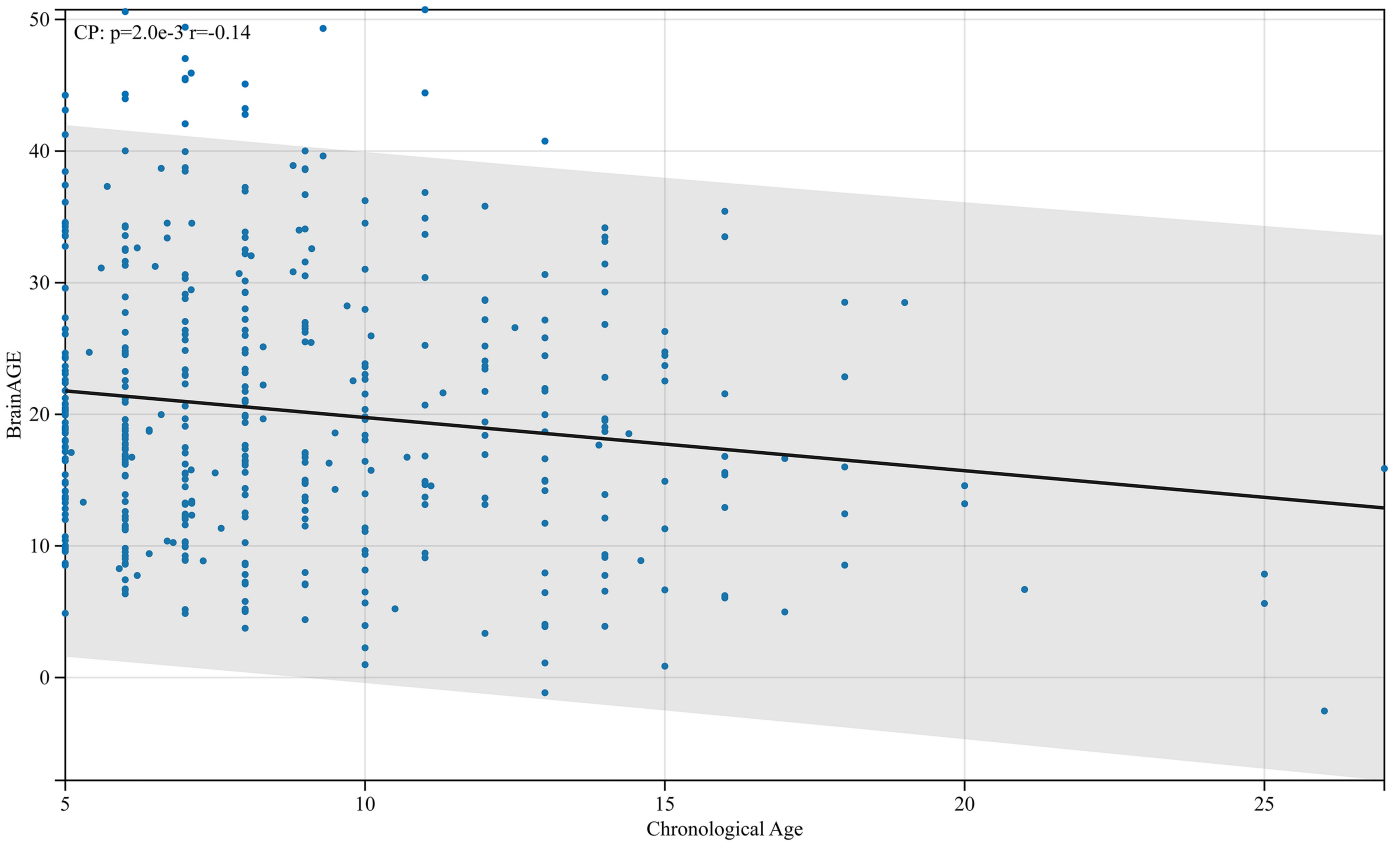
Relationship between BrainAGE difference and physiological age in patients with cerebral palsy. As shown in the figure, the BrainAGE of patients with CP negatively correlates with their physiological age (*r* = −0.14, *p* < 0.01).

From the analysis, the BrainAGE of children with CP aged 5 years compared with adults was negatively correlated with their physiological age (Figure 8). This implies that as children with CP grow older, the difference between cerebral and physiological age reduces. Children with CP after fetal or infantile brain damage caused by different factors have a corresponding decline in markers such as Tau protein after treatment (26). Even with treatment, the damage may continue to affect cerebral development in children with CP, and the effect peaks before age 5 and maintains a certain level. Therefore, the BrainAGE of children with CP aged 5 and above will gradually decrease with an increase in their age. This was consistent with the canalization concept described by Waddington (27).

In this study, we found that the BrainAGE of male patients with CP was higher than that of female patients (Figure 7B), and the number of male patients in the whole CP group was also higher than that of female patients. In CP, women are more tolerant of brain damage than men. This conclusion is consistent with the results of some clinical investigations and animal experiments (28, 29). This phenomenon can also be explained by the concept of canalization put forward by Waddington. From the fertilized egg, each cell group differentiates into different tissues, and each tissue has a great trend of specialization relative to other tissues. If this process deviates from its path for any reason, an undetermined regulatory process will immediately make the necessary corrections (27). In this process of regulation, women are better at regulation than men, which is true in humans and other mammals (30, 31). Women have a better ability to adjust their brain development back to the right track after brain damage than men.

This study reveals that the BrainAGE of spastic CP is higher than that of mixed CP (Figure 7C), and the BrainAGE of bilateral spastic CP is higher than that of unilateral spastic CP (Figure 7D). The BrainAGE of patients with nonasphyxia was higher than that of patients with asphyxia in female mixed CP (Figure 7E). These may be related to the MRI findings. First, the abnormal probability of MRI in spastic CP is higher than that in mixed CP. This may result in a higher BrainAGE in mixed CP than in spastic CP in some single samples, usually when spastic CP is mixed with other types of CP. However, because mixed CP can also be a mixture of the other two types of CP, this results in lower average values of BrainAGE in mixed CP than in spastic CP in larger sample sizes. Second, spastic CP is characterized by white matter injury and severe cortical atrophy. If the patient is spastic quadriplegia, the MRI manifestation is more serious and the prognosis is worse. Mixed CP refers to CP associated with more than two types of clinical manifestations. The location of the lesion is uncertain, and most of them show ventricular dilatation and cortical atrophy. Therefore, according to the rules of the brain map obtained by Bethlehem et al. (18), the average brain age of patients with spastic CP is higher than that of mixed CP.

In this study, a brain age prediction model was constructed without white and gray matter segmentation. The good performance of the model indicated that the brain age prediction model based on 2D-CNN was feasible. The brain age of children with CP was assessed using the brain age prediction model. Brain development in patients with CP over the age of 5 years was also examined. This study provides a research direction and basis for brain developmental abnormality studies in children with CP. Of course, our study also has some shortcomings. The study was designed as a cross-sectional study, and the conclusions may not be as convincing as a longitudinal cohort study. In the future, through collaborations, we will conduct a long-term longitudinal MRI follow-up of large samples of patients with CP to complete the next phase of the study.

## Conclusion

We observed that the 2D-CNN brain age prediction model without segmentation of white and gray matter can accurately predict the brain age of healthy people when the quality of head MRI data is poor. We also found that brain aging occurred in patients with CP. In patients with CP, the brain tolerance of female patients to damage factors is higher than that of male patients. The damage of spastic CP to brain development was higher than that of mixed CP, and the degree of brain aging in patients with spastic CP was higher than that in patients with mixed CP, and the brain aging in patients with spastic bilateral paralysis was more serious than that in patients with spastic unilateral paralysis.

## Data availability statement

The datasets presented in this article are not readily available because of ethical and privacy restrictions. Requests to access the datasets should be directed to the corresponding author.

## Ethics statement

The studies involving human participants were reviewed and approved by Medical Ethics Committee of the Second Affiliated Hospital of Xinjiang Medical University. Written informed consent from the participants' legal guardian/next of kin was not required to participate in this study in accordance with the national legislation and the institutional requirements.

## Author contributions

CZ contributed to experimental design, database registration, data analysis, drawing, and thesis writing. BY contributed to data collation and MRI data preprocessing. NM contributed to experimental supervision, guidance, and clinical information collation. YF contributed to MRI data processing and model training. JS contributed to MRI data collation. JW contributed to cerebral palsy MRI data preprocessing. QG contributed to cerebral palsy MRI data collation. SB contributed to clinical information collection. LT contributed to clinical information collection. XL contributed to experimental supervision, guidance, capital acquisition, and paper revision. All authors contributed to the article and approved the submitted version.

## Funding

This study was supported by grants from the Tomorrow Plan for Surgical Rehabilitation of Disabled Orphans of the Ministry of Civil Affairs of the People's Republic of China, the Rehabilitation Program for disabled Children of the Autonomous region disabled Persons' Federation and the Regional Project of the National Natural Science Foundation of China [81660202].

## Conflict of interest

The authors declare that the research was conducted in the absence of any commercial or financial relationships that could be construed as a potential conflict of interest.

## Publisher's note

All claims expressed in this article are solely those of the authors and do not necessarily represent those of their affiliated organizations, or those of the publisher, the editors and the reviewers. Any product that may be evaluated in this article, or claim that may be made by its manufacturer, is not guaranteed or endorsed by the publisher.

## Footnotes

^1^CP, Cerebral palsy.

^2^SPR, Selective posterior rhizotomy.

^3^CPVS, Cervical perivascular sympathectomy.

^4^Magnetic resonance image.

^5^3D-CNN, Three-dimensional convolution neural network.

^6^BrainAGE, Brain age gap estimation.

^7^Montreal Neurological Institute.

^8^Two-dimensional convolution neural network.

^9^Magnetic resonance images.

^10^Mean squared error.

^11^Mean absolute error.

^12^Determination coefficient.

^13^Root-mean-squared error.
