# Supplementary material for: Predicting the brain age of children with cerebral palsy using a two-dimensional convolutional neural networks prediction model without gray and white matter segmentation
Source: Front Neurol. 2022 Nov 24;13:1040087. doi: 10.3389/fneur.2022.1040087 (PMC9730825; doi:10.3389/fneur.2022.1040087)
Supplement: Supplementary file 1 [file Table_1.DOCX]

**Supplementary Table S1. Data sources for healthy brain age training sample**

| Cohort | N | Age  mean (SD) | Age  range | Sex  male/female | Repository details | | Scanner  (Field strength) | | Scan | Voxel dimensions | |
| --- | --- | --- | --- | --- | --- | --- | --- | --- | --- | --- | --- |
| Autism Brain Imaging Data Exchange (ABIDE^[[1]](#footnote-1)^) | 1179 | 16 (8.58) | 5-64 | 897/282 | INDI | | Various (all 3T) | | MPRAGE | Various | |
| Beijing Normal University (Enhanced Simple) | 180 | 23 (1.94) | 17-28 | 73/107 | INDI | | Siemens (3T) | | MPRAGE | 1.33x1.0x1.0 | |
| LEMOM | 39 | 29.59(8.38) | 20-49 | 18/21 | INDI | | Siemens (3T) | | MPRAGE | 1.0x1.0x5.0 | |
| ADHD200 | 585 | 11.64 (3.51) | 7-26 | 306/279 | INDI | | Various (3T) | | 3D IR-FSPGR | 1.0x1.0x1.0 | |
| Cleveland Clinic | 31 | 43.55 (11.14) | 24-60 | 11/20 | INDI | | Siemens Tim Trio (3T) | | MPRAGE | 2.0x1.0x1.2 | |
| International Consortium for Brain Mapping (ICBM^[[2]](#footnote-2)^) | 86 | 44.19 (17.92) | 19-85 | 41/45 | LONI IDA^[[3]](#footnote-3)^ | | Siemens Magnetom (1.5T) | | MPRAGE | 1.0x1.0x1.0 | |
| Information eXtraction from Images (IXI^[[4]](#footnote-4)^) | 562 | 48.64 (16.49) | 19-86 | 250/312 | http://biomedic.doc.ic.ac.uk/brain-development | | Philips Intera (3T); Philips Gyroscan Intera (1.5T); GE Signa (1.5T) | | T1-FFE; MPRAGE | 0.9375x0.93751x1.2 | |
| Consortium for Reliability and Reproducibility (CoRR^[[5]](#footnote-5)^) | 488 | 23.06 (10.58) | 6-62 | 252/236 | INDI | | Siemens Tim Trio (3T);GE Discovery MR750(3T) | | Various | Various | |
| The Center for Biomedical Research Excellence (COBRE^[[6]](#footnote-6)^) | 74 | 35.82 (11.58) | 18-65 | 51/23 | INDI | | SIEMENS Tim Trio (3T) | | MPRAGE | 1.0x1.0x1.0 | |
| Wayne State University(10,11,EF Dataset) | 319 | 52.72 (18.05) | 18-83 | 100/219 | INDI | | Siemens Magnetom (1.5T) | | MPRAGE | Various | |
| Parkinson's Disease Datasets | 36 | 66.03 (8.92) | 46-82 | 16/20 | INDI | | Siemens Tim Trio (3T); Siemens Avanto(1.5T) | | MPRAGE(IR) | 1.0x1.0x1.0; 0.97x0.97x1.0 | |
| MEDICS（SIMON Dataset） | 73 | 43.47 (3.60) | 29-46 | 73/0 | INDI | | Various (3.0T) | | MPRAGE | 1.0x1.0x1.0 | |
| WUSL (Power, 2012) | 77 | 14.77 (6.21) | 6-24 | 37/40 | INDI | | Siemens Tim Trio (3T) | | MPRAGE | 1.0x1.0x1.0 | |
| Child Mind Institute Healthy Brain Network (CMI-HBN^[[7]](#footnote-7)^) | 6 | 5.17 (0.41) | 5-6 | 3/3 | INDI | | Siemens (3T) | | MPRAGE | 1.0x1.0x1.0 | |
| Brain-Age Healthy Control Dataset | **3735** | **26.93 (19.03)** | **5-86** | **2128/1607** | | **-** | | **-** | | **-** | **-** |
| INDI (<http://fcon_1000.projects.nitrc.org>)  LONI ([https://ida.loni.usc.edu)](about:blank)  ABIDE Dataset, ADHD200 Dataset and CoRR Dataset all comprising data from various sites with different scanners/parameters | | | | | | | | | | | |

1. Autism Brain Imaging Data Exchange [↑](#footnote-ref-1)
2. International Consortium for Brain Mapping [↑](#footnote-ref-2)
3. Laboratory of Neuro Imaging Image & Data Archive [↑](#footnote-ref-3)
4. Information eXtraction from Images [↑](#footnote-ref-4)
5. Consortium for Reliability and Reproducibility [↑](#footnote-ref-5)
6. The Center for Biomedical Research Excellence [↑](#footnote-ref-6)
7. Child Mind Institute Healthy Brain Network [↑](#footnote-ref-7)
